# Improving biocide evaluation using propidium monoazide (PMA) viability staining technique

**DOI:** 10.1038/s41598-025-32251-z

**Published:** 2025-12-19

**Authors:** Xiang Shi, Hasrizal Abd Rahman, Julia R. de Rezende

**Affiliations:** 1 Lyell Centre, Heriot-Watt University, Edinburgh, UK; 2 James Watt School of Engineering, University of Glasgow, Glasgow, UK; 3 PETRONAS Research Sdn. Bhd., Bandar Baru Bangi, Malaysia

**Keywords:** Biocide evaluation, Propidium monoazide (PMA), Viability staining, Biological techniques, Biotechnology, Environmental sciences, Microbiology

## Abstract

Chemical biocides are commonly employed to manage problems caused by microbial processes. In the energy sector, for example, engineered systems are often treated with biocides to control microbiologically influenced corrosion (MIC), biofouling, and the biological generation of hydrogen sulfide. Standard DNA-based methods that are widely used to assess biocide effectiveness often cannot distinguish between live and dead microorganisms, potentially leading to inflated estimates of living cell populations. Incorporating propidium monoazide (PMA) viability staining technique offers a promising solution to this limitation. In this study, we explored the application of PMA within a standard DNA-based workflow to evaluate biocide performance more accurately. A model sulfate-reducing microbial consortium, derived from oilfield produced water, was exposed to widely used biocides including glutaraldehyde (Glut) and tetrakis(hydroxymethyl)phosphonium sulfate (THPS). PMA was applied prior to standard DNA extraction and subsequent qPCR and amplicon sequencing procedures. We observed PMA-derived microbial abundance at least an order of magnitude lower compared to that without PMA. The reduced PMA-derived microbial abundance correlated with the lower ability of the model microbial communities to produce hydrogen sulfide – an association that was absent based on the usual approach without PMA. Biocide-treated communities, in comparison to untreated controls, displayed significant alterations in their microbial ecological properties, such as alpha diversity, beta diversity, and taxonomic composition, as determined through 16S rRNA gene sequencing – differences that were only apparent when PMA was applied. These results confirm that incorporating PMA into standard DNA-based biocide assessment protocols is both feasible and beneficial. Since PMA implementation requires minimal additional effort, we advocate for its adoption in future biocide performance studies, in particular for engineered systems in the energy industry.

## Introduction

Microbial communities are prevalent across a range of engineered systems in the energy sector, including those used for oil and gas extraction, geothermal energy operations, and underground carbon dioxide storage^1–3^. If not properly managed, microbial proliferation in these environments can cause significant operational challenges, such as degradation of hydrocarbon products, microbiologically influenced corrosion (MIC), reduced heat exchange efficiency, obstruction of flow, biofouling, and the formation of unwanted byproducts like hydrogen sulfide^4–8^. To prevent or mitigate these effects, chemical biocides are routinely used to eliminate microbes and inhibit their activity^9^. Among the most commonly deployed biocides in the energy sector are glutaraldehyde (referred to hereafter as Glut) and tetrakis(hydroxymethyl)phosphonium sulfate (referred to as THPS)^10–15^. Biocide efficacy is traditionally assessed using “bottle tests,” as outlined in the AMPP standard TM0194-2014. In this method, representative microbial samples, typically sourced from field environments previously exposed to biocide treatments, are placed in test microcosms and subjected to serial dilutions. These samples are then incubated for up to 28 days. Microbial presence is determined either qualitatively (positive/negative growth) or quantitatively using the most probable number (MPN) technique. Biocide performance is evaluated based on differences in microbial growth across the dilution series. Despite its widespread use and acceptance, this methodology has been criticized for being labor-intensive and for its inability to detect microorganisms that cannot be cultured in the laboratory^7,16^.

The growing affordability of sequencing technologies and recent developments in molecular microbiology have contributed to the increased adoption of DNA-based techniques, such as quantitative PCR (qPCR) and amplicon sequencing, in evaluating biocide performance. These molecular tools eliminate the need for culturing microorganisms, a key step in traditional “bottle test” methods, by enabling direct analysis of microbial communities after biocide exposure. As a result, they serve as a valuable complement to conventional approaches, allowing for faster and more detailed insights into shifts in microbial abundance and community composition under biocidal pressure^17^. However, one significant limitation of DNA-based analyses is their inability to discriminate between live and dead microorganisms. This distinction is crucial, as only live microbes contribute to ongoing and future metabolic activity, biocide resistance, and community adaptation, all of which are central to assessing biocide effectiveness^18,19^. When a considerable portion of the microbial population is dead, for example after effective application of biocide, DNA assays can produce inflated estimates of active microbial load, potentially leading to unnecessarily high biocide dosages. Such overestimations not only increase treatment costs but also raise environmental concerns^20,21^. Moreover, this lack of differentiation complicates efforts to pinpoint the most biocide-resistant members of the community, thereby limiting the ability to optimize treatment strategies based on microbial resilience and response patterns.

Several analytical methods, including ATP and RNA-based assays, have been developed to minimize the interference caused by non-viable microbial cells in microbial evaluations. ATP assays quantify adenosine triphosphate levels by leveraging a luciferase-mediated bioluminescent reaction, generating light measured in relative light units (RLU), which serves as a proxy for viable and metabolically active cells. Despite their utility, ATP assays offer only a general indication of microbial activity and do not provide details about community structure, species diversity, or the dynamics of individual populations over time^21–23^. RNA-based approaches attempt to target the living fraction of a microbial community by detecting actively transcribed RNA molecules, primarily rRNA and mRNA^21^. However, these methods face practical hurdles, as RNA is highly susceptible to degradation during sampling and processing, especially in low-biomass environments such as those treated with biocides, making it difficult to obtain RNA of sufficient quantity and quality^24^.

Propidium monoazide (PMA) viability staining technique offers a compelling alternative that addresses these limitations effectively. PMA is a membrane-impermeable dye that selectively penetrates cells with compromised membranes, typically dead cells, and binds to their DNA. Upon exposure to strong light, the dye forms covalent bonds with DNA, rendering it unamplifiable in downstream molecular analyses. In contrast, DNA from cells with intact membranes remains unaffected and can be amplified using techniques such as qPCR or amplicon sequencing. This allows for a more accurate reflection of the viable microbial population. For optically clear samples that allow light to pass through, the PMA approach is not only reliable and easy to implement, but it also integrates seamlessly into standard DNA-based workflows. Since it differentiates between intact (live and potentially active) and damaged (dead) cells based on membrane integrity, it enhances the resolution of microbial viability assessments in biocide performance studies^25,26^.

PMA-based viability staining technique has been widely applied and validated across diverse settings and sample types. For instance, PMA has been employed to detect and measure viable pathogens and infectious viruses associated with human illnesses, as well as microbial contaminants responsible for food spoilage during processing^27–33^. In environmental applications, PMA has been used to assess the presence of living bacteria in wastewater treatment systems^34^. Researchers have also developed and refined PMA-based diagnostic protocols to more accurately identify viable bacterial populations in clinical samples such as urine and vaginal microbiota^35,36^. In agricultural contexts, PMA has enabled the detection of live seed-borne pathogens linked to plant diseases and the monitoring of harmful viruses in irrigation water^33,37^. PMA has also proven valuable in ecological studies for examining how environmental variables shape the composition and diversity of viable microbial populations in soils, sterile environments like cleanrooms, and even within human respiratory and gastrointestinal tracts^38–41^. The technique has also been used to explore microbial stress responses – for example, studying how DNA functions during desiccation conditions^42^; and to assess how extracellular DNA can influence microbial community profiling in marine sediments^43^. Additionally, PMA has supported research into enhancing biodiversity monitoring by improving the selectivity and sensitivity of environmental DNA (eDNA) analyses^44^.

Despite its extensive use across various fields, there is currently no documented research to our knowledge focused specifically on applying PMA for evaluating biocide performance in engineered systems within the energy sector – such as oil and gas production, geothermal energy extraction, or underground CO₂ storage. While insights from previous PMA applications provide a useful foundation, they cannot be directly translated to these settings due to the distinctive characteristics of the samples involved. In this context, microbial communities are exposed to a complex combination of harsh environmental conditions, including limited oxygen availability, elevated temperatures and pressures, high salinity, and the presence of chemical biocides^20^. These stressors may lead to physiological adaptations in microorganisms, such as alterations in membrane permeability, surface chemistry, enzymatic functions, or the activity of efflux mechanisms. These adaptations could, in turn, influence how effectively PMA differentiates between live and dead cells^45–47^. Furthermore, the situation is complicated by the properties of certain widely used biocides. For instance, glutaraldehyde (Glut), commonly applied in concentrations ranging from 50 to 2000 ppm in industrial biocidal treatments, is also used in much higher concentrations (typically above 10,000 ppm or 1%) as a fixative in laboratory protocols. In these higher concentrations, Glut acts to stabilize and preserve cell structure for downstream analyses such as microscopy or flow cytometry, effectively preventing cell lysis^48,49^.

This study aimed to assess the feasibility and added value of incorporating the PMA-based viability staining technique into biocide performance evaluations, specifically within engineered environments in the energy sector. Our investigation was centred on determining whether integrating PMA with standard DNA-based techniques and microbial community analyses could enhance the reliability of biocide assessments. To this end, we cultivated a sulfidogenic microbial community sourced from produced water collected from an oil and gas reservoir and used the enriched community as the model microbial community for the study. This model community was subjected to four distinct treatments: glutaraldehyde (Glut), THPS, thermal treatment, and a no-treatment control. DNA was then extracted from each group, both with and without prior PMA treatment, and multiple microbial ecological metrics were examined, including microbial abundance, alpha diversity, beta diversity, and taxonomic composition. These metrics are commonly employed in microbial ecology studies within the energy industry and are increasingly recognized as valuable indicators of biocide efficacy^18,19^.

In addition, we explored the extent to which these ecological metrics, measured with and without the use of PMA, aligned with the microbial community’s hydrogen sulfide production following biocide application. Since our model community was dominated by sulfate-reducing microorganisms (SRM) that showed both high relative abundance and active sulfide generation, sulfide output served as a practical proxy for microbial metabolic activity. Given the operational and safety challenges posed by hydrogen sulfide in industrial systems, establishing a link between microbial community structure and functional output was critical to understanding the contribution of PMA-based viability assessments to more accurate and meaningful biocide evaluations.

## Materials and methods

### Preparation of microbial enrichment from the produced water

**Fig. 1:**
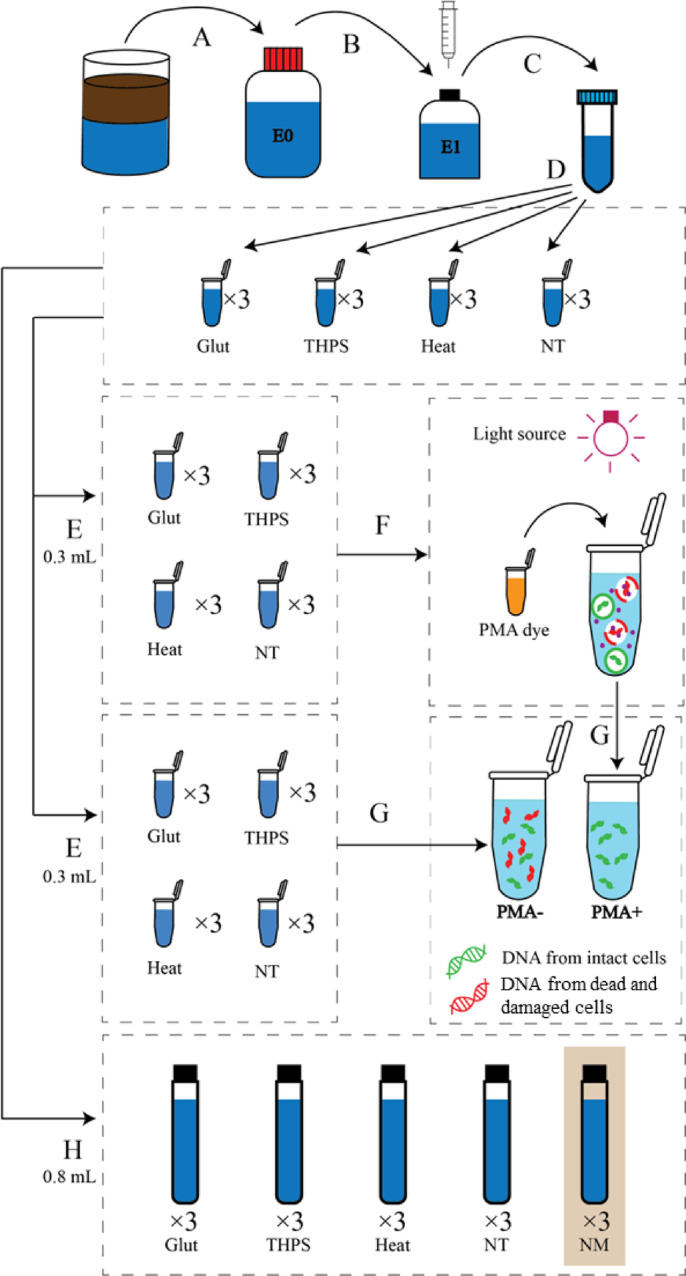
Workflow of the experiment until DNA extraction. (**A**) A SRM enrichment culture was set up using the oilfield produced water as the inoculum; (**B**) The E0 enrichment was later transferred to a freshly prepared medium to acquire an active microbial community (E1 enrichment); (**C**) Cell pellets were obtained from the active E1 enrichment, washed and re-suspended; (**D**) Cell resuspensions were treated with glutaraldehyde, THPS, heat or no treatment (NT); (**E**) 0.6 mL of cell suspensions from each 1.5 mL centrifuge tube of the four treatment groups were split into two 0.3 mL fractions (in triplicates) and transferred to 0.5 mL translucent centrifuge tubes; (**F**) One set of triplicate 0.3 mL fractions for each cell suspension underwent the PMA viability assessment (PMA+); (**G**) DNA was extracted from all cell suspensions, with or without the PMA viability assessment (PMA+ or PMA-, respectively). Expectedly, only DNA from intact cells was obtained from the fraction that underwent the PMA viability assessment, while DNA from both intact and damaged (dead) cells, was obtained from the fraction that did not undergo the PMA viability assessment; H: 0.8 mL of cell suspensions from each 1.5 mL centrifuge tube of the four treatment groups were transferred to Hungate tubes as inoculum for incubation, thus verifying the microbial sulfide production ability after the cell treatments. NM refers to the negative, medium-only control for this step of the experiment.

A field sample consisting of produced water and crude oil was collected from an oil-producing well in Malaysia. The sample was transferred into a metal drum with minimal headspace and transported at ambient temperature to Heriot-Watt University in Edinburgh, UK. Upon arrival, the container was left undisturbed in a dark environment at room temperature for 24 h to allow for phase separation between the produced water and crude oil. The subsequent microbial enrichment used in the core experiments was derived from the aqueous (produced water) phase. This enrichment was initiated by setting up a sulfate-reducing microbial culture in a 500 mL glass bottle sealed with a butyl rubber stopper (Step A, Fig. 1). For the culture setup, 25 mL of the produced water was combined with 475 mL of a modified anaerobic synthetic seawater medium^50^. The medium was altered from the original formulation by substituting 0.2 M sodium sulfide (Na₂S) with 0.2 M ascorbic acid as the reducing agent and by supplementing it with additional carbon sources (5 mM each of acetate, propionate, and butyrate). The bottle’s headspace was flushed and filled with a nitrogen/carbon dioxide gas mixture (90:10). This enrichment, termed E0, was incubated at 25 °C for 12 weeks and subsequently stored at 4 °C for later use.

### Preparation of active cell suspensions for the experiment and measurement of the sulfide concentration

Before initiating the main experiment, the E0 enrichment culture was transferred into a newly prepared medium to further enrich sulfate-reducing microorganisms (SRM). To begin, the E0 culture was gently inverted and lightly agitated to ensure even mixing. Then, using a syringe flushed with nitrogen gas to maintain anaerobic conditions, 20 mL of the homogenized culture was extracted and introduced into a 200 mL serum bottle containing 180 mL of the same modified anoxic synthetic seawater medium used in the E0 stage (Step B, Fig. 1). The bottle was sealed with a butyl rubber stopper, and the headspace was purged with a nitrogen/carbon dioxide mixture (90:10). This secondary enrichment, referred to as E1, was incubated at 25 °C for a period of 10 days.

Throughout the incubation, dissolved sulfide levels were measured as a proxy for microbial activity. To do this, the bottle was mixed by hand, and a 0.5 mL aliquot was withdrawn using a syringe. A 0.1 mL portion of this sample was promptly mixed with 1.9 mL of copper reagent solution, comprising 50 mM HCl and 5 mM CuSO₄. The absorbance of the resulting copper sulfide colloid was measured at 480 nm, and sulfide concentrations were calculated using a previously established calibration curve^51^. The detection threshold for sulfide concentration was 0.1 mM. After 10 days, the culture displayed a sulfide production rate of 0.8 mM/day, confirming its biological activity.

Following this enrichment phase, the E1 culture was processed to obtain active cell suspensions for use in downstream experiments. The serum bottle was opened, and its contents were immediately dispensed into four 50 mL centrifuge tubes (Fisherbrand, UK; Step C, Fig. 1). These were centrifuged at 4696×g (maximum speed; Heraeus Multifuge X1R, Thermo Fisher Scientific, UK) for 10 min at ambient temperature to pellet the cells. The supernatant was discarded, and the pellets were washed three times with phosphate-buffered saline (PBS, pH 7.2) to eliminate residual medium. Each wash involved resuspending the pellet in 10 mL of PBS, vortexing for 10 s, followed by centrifugation under the same conditions and discarding the supernatant. After the final wash, the pellets were resuspended in 5 mL of PBS per tube, and the contents of all four tubes were combined to produce a unified active cell suspension derived from the E1 enrichment.

### Biocide treatments for the cell suspensions

For the main experimental setup, the prepared cell suspension was aliquoted into twelve 1.5 mL microcentrifuge tubes (Eppendorf, UK; Step D, Fig. 1), forming four treatment groups with three replicates each. Two groups were assigned for biocide exposure – one for Glut and the other for THPS. In these groups, each tube was treated with either Glut or THPS at a final concentration of 200 ppm and incubated in the dark at ambient temperature for four hours to induce cell death.

A separate group served as the heat-killed control (Heat; Fig. 1). These tubes were heated at 95 °C for 10 min using a heat block (Eppendorf, UK), then held at room temperature in the dark for an additional four hours, consistent with the biocide-treated groups. The final group, which received no treatment (NT; Fig. 1), was maintained in the dark at room temperature for the same four-hour period.

Following treatment, all twelve tubes were centrifuged simultaneously at 10,000×g for 5 min at room temperature. After centrifugation, the supernatant was removed, and the pellets were subjected to three washing steps with 1.5 mL phosphate-buffered saline (PBS, pH 7.2), as previously described, to eliminate any remaining biocide or heat treatment residues. After the final wash, the pellets in each tube were resuspended in 1.5 mL of PBS.

### PMA application and DNA extraction after the treatment

Following the treatment of the cells, 0.6 mL of the suspension from each 1.5 mL microcentrifuge tube was taken and split into two equal 0.3 mL portions. These aliquots were placed into individual 0.5 mL translucent PCR tubes (Axygen, UK; Step E, Fig. 1). One of the two fractions from each sample was subjected to PMA staining using PMAxx™ (Biotium, UK). The dye was added to each designated PCR tube to achieve an approximate final concentration of 26 µM.

After dye addition, the tubes were incubated in the dark at room temperature for 10 min. This was followed by a 15-minute light activation phase under 15,000-lumen LED lights (Hargitech, UK; Step F, Fig. 1). During both the incubation and light exposure steps, the tubes were manually inverted and gently shaken by hand for 5 s every minute. After photoactivation, the treated contents were transferred into new 2 mL microcentrifuge tubes.

Meanwhile, the second 0.3 mL fraction from each sample was transferred directly into separate 2 mL microcentrifuge tubes without PMA treatment and held at room temperature for no longer than an hour. All tubes, whether treated with PMA or not, were then centrifuged at 10,000×g for 5 min to collect cell pellets, and the supernatants were removed. Each pellet was washed three times using 1.5 mL of PBS (pH 7.2), following the same protocol described previously.

DNA was then extracted from all resulting PBS-washed pellets using a cetyltrimethylammonium bromide (CTAB)-based extraction method, described in detail elsewhere^52^, This process yielded a total of 36 DNA samples for subsequent analysis (Step G, Fig. 1).

### Verification of microbial sulfide production activity after the biocide treatments

To assess the sulfide-producing capacity of the microbial community following the treatments, cell suspensions from the biocide-treated groups, the heat-treated group (serving as a non-biocide control), and the no-treatment control group were incubated in sulfate-reducing conditions, and the changes in dissolved sulfide concentrations were monitored. In brief, 0.8 mL of cell suspension from each of the twelve 1.5 mL microcentrifuge tubes was transferred to Hungate tubes, which were sealed with butyl rubber stoppers and filled with 10 mL of the same modified anoxic synthetic seawater medium used in the E0 enrichment (Step H, Fig. 1). The headspace of each Hungate tube was replaced with a 90:10 mixture of N2/CO2. Negative control groups, consisting solely of medium, were also prepared in triplicate (NM; Fig. 1). The tubes were incubated at 25 °C for 28 days, and sulfide concentrations were measured at both the start and end of the incubation period to evaluate the sulfide production potential of the microbial communities.

### qPCR targeting 16S rRNA genes

To estimate the total microbial abundance from DNA extracts obtained in Step G (Fig. 1), quantitative PCR (qPCR) targeting 16S rRNA genes was performed using the primer pair Ba519F - CAGCMGCCGCGGTAANWC and Ba907R - CCGTCAATTCMTTTRAGTT^53^. The reactions were carried out on a QuantStudio™ 3 Real-Time PCR machine (Thermo Fisher Scientific, US) in a 10 µL system, which included 5 µL of SsoAdvanced™ Universal SYBR^®^ Green Supermix (Bio-Rad, UK), 3 µL of template DNA, and 500 nM of each primer. The thermal cycling conditions were set as follows: initial denaturation at 96 °C for 2 min, followed by 40 amplification cycles of denaturation at 96 °C for 30 s, annealing at 52 °C for 30 s, and extension at 72 °C for 1 min. To confirm the specificity of the assays, melting curves were generated post-qPCR by gradually increasing the temperature from 60 °C to 95 °C in 0.2 °C increments (5 s per step). Each assay was run in technical triplicate for all standards, query templates, and no-template controls (NTC).

To account for discrepancies in amplification efficiency between standards and templates, one-point calibration method (OPC) was used to convert the raw data to the abundance of 16S rRNA genes^18,54,55^. The raw fluorescence data were imported into LinRegPCR (version 2018.0), where the threshold cycle number (Ct) and amplification efficiency (E) for each reaction were determined individually^56,57^. As described earlier^18^, the standard for 16S rRNA genes was prepared from an oilfield isolate *Sulfurimonas* sp., and the abundance of 16S rRNA genes in each query template was calculated using serial dilutions of the standard (10^4^–10^7^ genes/µL). The detection limit of the qPCR assay was set to 100 genes/µL, which corresponds to approximately 16,000 genes/mL of sample. The abundance of 16S rRNA genes was then converted into the number of cells, assuming an average of 5.4 gene copies per cell (https://rrndb.umms.med.umich.edu/, accessed May 2025)^58^. Finally, the cell counts were log10-transformed, and statistical differences in abundance between treatment groups were analyzed using analysis of variance (ANOVA) followed by Tukey’s HSD Post Hoc test.

### 16S rRNA gene sequencing and data analyses

The V4 region of the 16S rRNA gene was sequenced from all DNA samples extracted in Step G (Fig. 1). Library preparation and sequencing were carried out by NU-OMICS at Northumbria University (UK) using the MiSeq protocol outlined in previous studies^59^. The PCR reaction mixture contained 1× Accuprime Pfx Supermix, 0.5 µM of each primer, and 1 µL of template DNA. The PCR conditions were as follows: initial denaturation at 95 °C for 2 min, followed by 30 cycles of 95 °C for 20 s, 55 °C for 15 s, and 72 °C for 5 min, with a final extension at 72 °C for 10 min. The primer pair used was F515 - GTGCCAGCMGCCGCGGTAA and R806 – GGACTACHVGGGTWTCTAAT^60^. Based on TestPrime (http://www.arb-silva.de/search/testprime) and the SILVA small subunit (SSU) database release 138 (last accessed May 2025), the chosen primer pair targets 94.5% of archaeal and 92.7% of bacterial 16S rRNA gene sequences with one allowed mismatch^61^. PCR product concentrations were measured using the Quant-iT™ PicoGreen™ dsDNA Assay (Invitrogen), and each sample was normalized to 10 nM. All samples from a 96-well plate were pooled and quantified by fragment size using the BioAnalyzer (Agilent Technologies) and concentration determined by Qubit (Invitrogen). The pools were combined in equimolar amounts to form a single library, which was denatured with 0.2 M NaOH for 5 min and diluted to 5 pM with 15% PhiX before being loaded onto a MiSeq V2 500 cycle cartridge.

Amplicon sequencing data were processed using mothur version 1.48^62^, with a workflow adapted from the MiSeq SOP (https://mothur.org/wiki/miseq_sop/, accessed in Mar 2025), and incorporating slight procedural adjustments^61^. Initially, paired-end reads were merged into contiguous sequences using quality score-based alignment. These contigs were screened to retain sequences not exceeding 275 base pairs. Subsequently, all unique sequences were aligned against the SILVA v138.1 reference alignment from the mothur repository (https://mothur.org/wiki/Silva_reference_files#Release_138). Taxonomic classification was assigned to amplicon sequence variants (ASVs), retaining only those attributed to archaeal and bacterial domains. Shannon indexes (representing alpha diversity) were computed for each sample with all samples normalized to a sequencing depth of 4937 reads through rarefaction. This same rarefied dataset (4937 reads per sample) was used for beta diversity assessments. ASVs were grouped at the genus level, and Bray-Curtis dissimilarities were computed to evaluate compositional differences between samples. Visualisation of sample relationships was performed using non-metric multidimensional scaling (NMDS) plots created via the phyloseq R package^63^. For broader comparison, the mean genus-level abundance was calculated for each treatment condition. Jaccard distances were then determined between treatment groups based on the aggregated taxonomic profiles, using the vegan package in R^64^. All statistical procedures were performed in R version 4.2.2. Group-level comparisons of alpha diversity (Shannon index) were assessed using one-way ANOVA, followed by Tukey’s HSD for pairwise contrasts. To examine treatment group clustering in the NMDS plots, PERMANOVA tests were conducted using the adonis function in the vegan package.

## Results

### Abundance and sulfide production activity of the microbial community

In this study, our primary focus was to explore whether supplementing the established DNA assays and microbial community analyses with the PMA viability assessment could be beneficial for the evaluation of biocide performance, particularly for biocide treatments in engineered system across the energy industry, with minimal change to established DNA-based protocols. The implementation of the PMA viability assessment aimed to address the influence of DNA from dead cells within the biocide-influenced microbial communities, thus enhancing the accuracy of biocide evaluation. For clarification, in this study DNA from dead cells refers to DNA from dead and damaged cells with compromised membranes and free DNA that is no longer enclosed by a membrane.

**Fig. 2:**
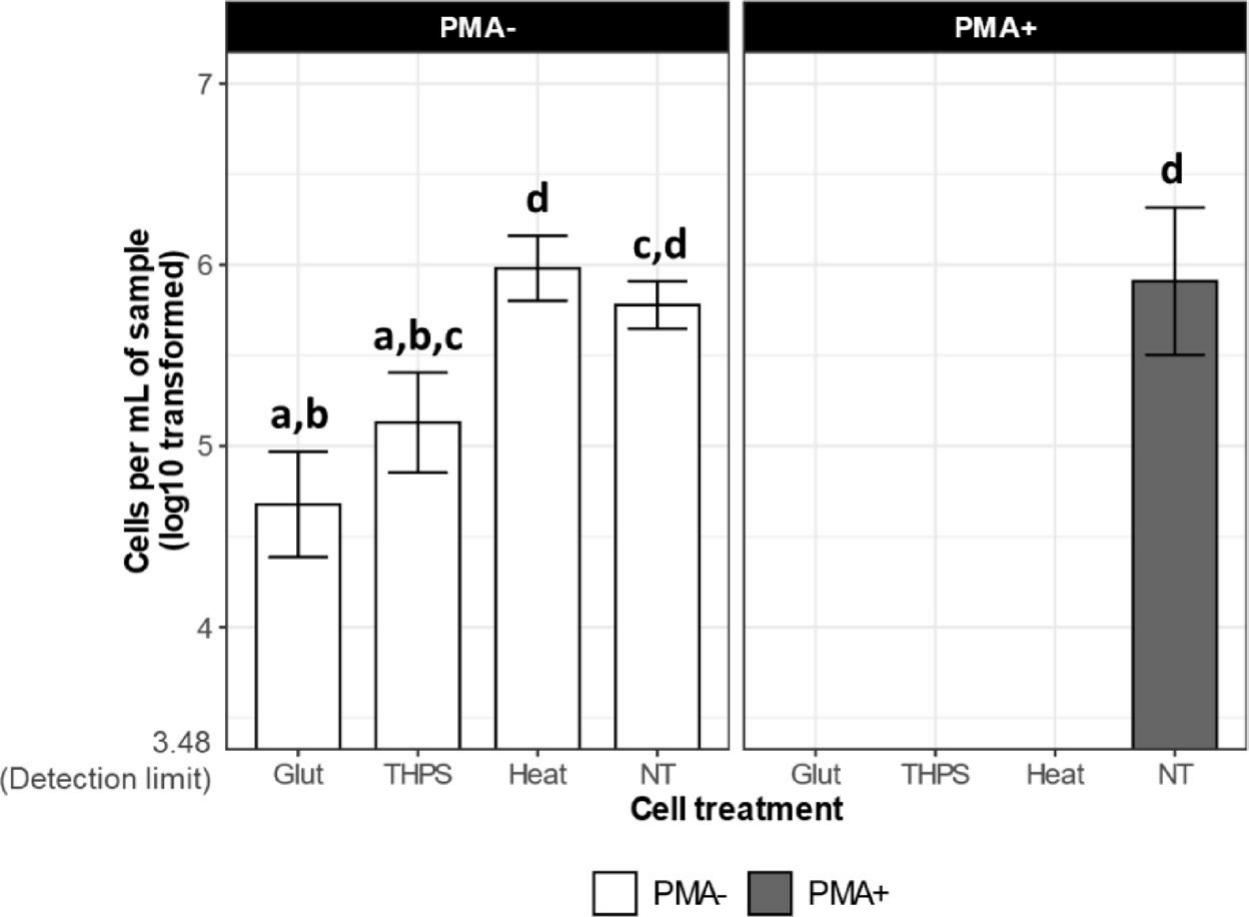
Microbial abundance estimated from qPCR targeting 16S rRNA genes. The samples were categorised as PMA- or PMA+, indicating PMA was not/was applied before DNA extraction, respectively. The error bars represent the standard deviation among the three biological replicates. Bars annotated with the same letter indicate no significant difference in the mean cells per mL of sample between the treatment groups they represent, as determined by analysis of variance (ANOVA) and Tukey’s HSD Post Hoc test.

We began our investigation by comparing the *microbial abundance* within samples subjected to biocides (Glut and THPS), heat (Heat), or no treatment (NT). The samples received or did not receive PMA prior to DNA extraction (PMA+ or PMA-, respectively). Without PMA, the microbial abundance appeared significantly lower in Glut (5.0 × 10^4^ cells/mL; Fig. 2) compared to Heat (1 × 10^6^ cells/mL) and NT (6.3 × 10^5^ cells/mL; p_adjust_<0.01 in both cases). The microbial abundance in THPS (1.3 × 10^5^ cells/mL) was also significantly lower than Heat (p_adjust_<0.05), but not significantly different from NT. The microbial abundance was not affected in Heat compared to NT (Fig. 2). When PMA was applied before DNA extraction to remove DNA from dead cells, it revealed that the microbial abundance was below the detection limit for all killing methods (Glut, THPS and Heat), representing at least 1.2, 1.6 and 2.6 log10-fold further reduction compared to PMA- samples. The microbial abundance of the NT group was similar between PMA+ (8.1 × 10^5^ cells/mL) and PMA- samples (6.3 × 10^5^ cells/mL), indicating PMA itself did not affect the microbial abundance of this control group, which is expectedly dominated by live cells (Fig. 2). Overall, when compared to NT, our results suggest that Glut and THPS showed an evident reduction in microbial abundance even without PMA-mediated signal suppression of membrane-compromised cells, with the reduction resulting from Glut being more substantial than THPS. However, with PMA, the reduction in microbial abundance by either biocide was revealed to be more striking than it appeared without PMA. In addition, the reduction in microbial abundance caused by Heat could only be detected when DNA from the dead cells was removed.

**Fig. 3:**
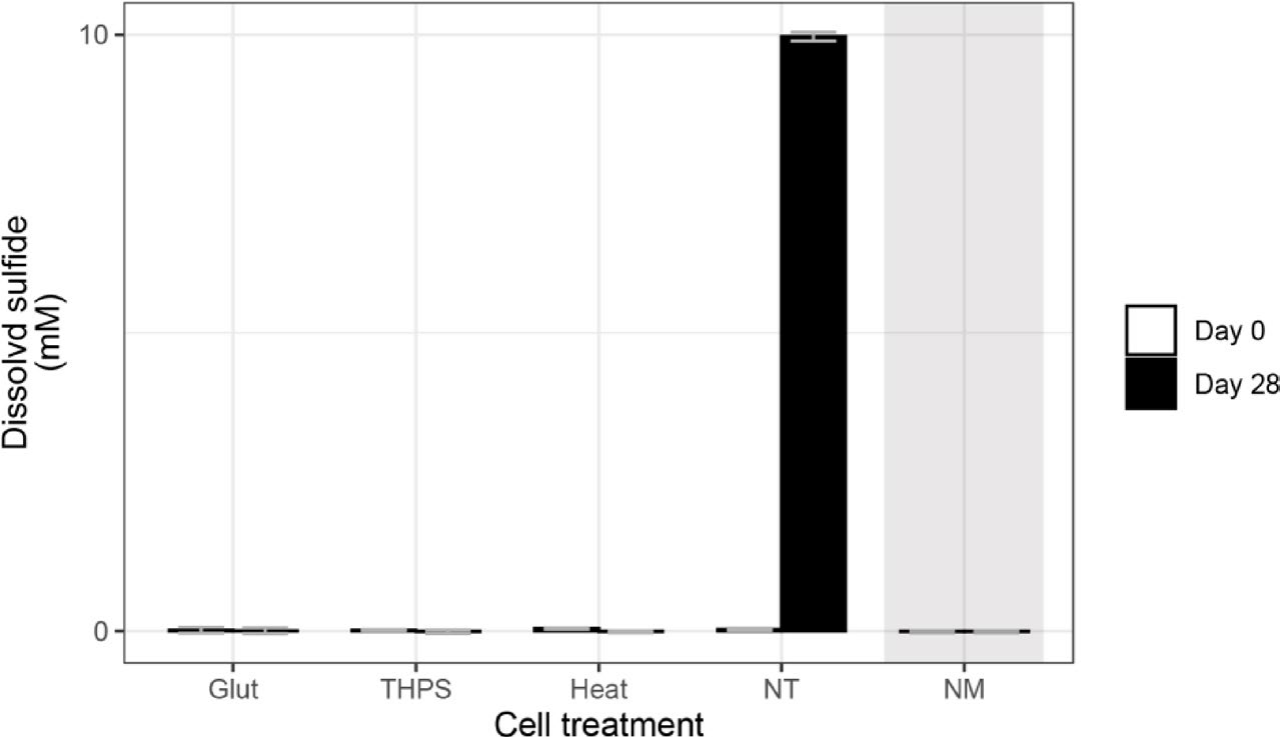
Concentration of dissolved sulfide. The concentration of dissolved sulfide was measured at the beginning and end of the 28-day incubation period. Bars represent the average concentration among the biological replicates from each treatment and control groups (*N* = 3). Error bars indicate the standard deviation among the biological triplicates. The shaded panel indicates the medium-only control (NM).

In order to examine the relationship between the abundance of the microbial community and its metabolic activity of sulfide production following different cell treatments (referred to as step D in Fig. 1), we monitored the dissolved sulfide concentration after a 28-day incubation experiment subsequent to the cell treatments (referred to as step H in Fig. 1). By the end of the 28-day incubation, the dissolved sulfide concentration of the biocide-treated groups (Glut and THPS) and the Heat group showed no significant change compared to day 0, measuring below the detection limit of 0.1 mM (Fig. 3). In contrast, the positive control group (NT) exhibited an increase in dissolved sulfide concentration from ca. 0 mM on day 0 to ca. 10 mM by day 28. As expected, the medium-only control (NM) maintained a sulfide concentration below the detection limit at both time points. This indicates that microbial sulfide production was suppressed by both the biocides and heat treatments and correlated with the abundance profiles revealed by the PMA+ samples, and not with the PMA- samples (Fig. 3).

### Alpha and beta diversity, and taxa distribution of the microbial community

**Fig. 4:**
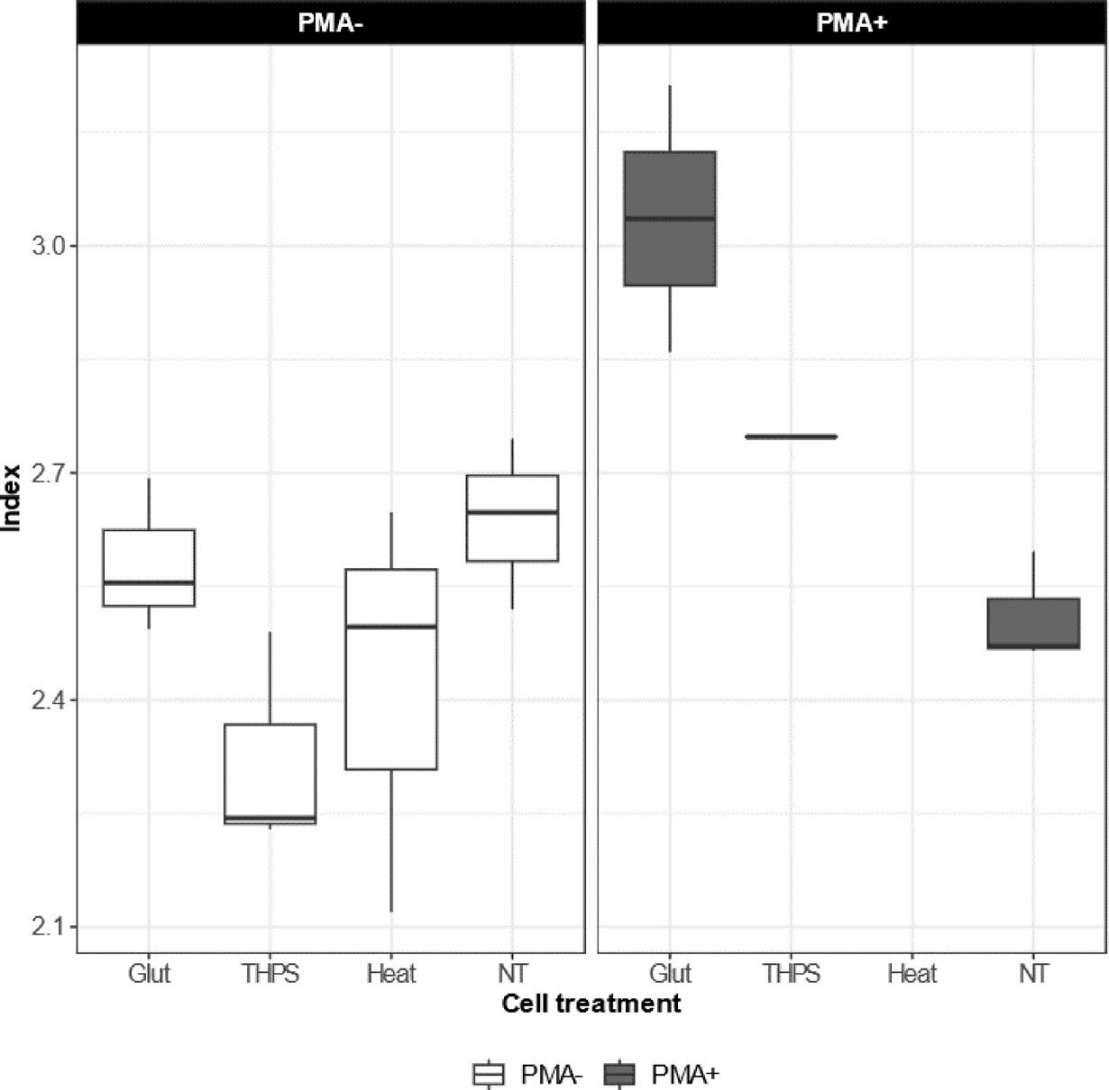
Shannon index for samples from different treatment groups. Shannon index estimation was only available for samples from which more than 4937 unique sequences were identified, i.e. all PMA- samples and NT PMA+ samples (*N* = 3), 2 of the Glut PMA+ samples (*N* = 2), and 1 of the THPS PMA+ samples (*N* = 1).

Next, we investigated how PMA would affect the *alpha diversity* of the microbial communities across different treatment groups. We chose the Shannon index as the representation of alpha diversity^65^. The Shannon index offers a measure of alpha diversity by considering the number of different species present in the sample (i.e. richness) and the distribution of species abundance within the sample (i.e. evenness). Without PMA, the Shannon indices for all the PMA- samples varied between ca. 2.1 and 2.7 (Fig. 4). No statistically significant differences were detected among the four groups (Glut, THPS, Heat, NT). With PMA, the Shannon indices for the PMA+ samples from the NT group remained similar to those of the PMA- samples for this group, which again shows no influence of PMA on a group dominated by live cells. For the PMA+ samples from the Glut and THPS groups, the Shannon indices increased to ca. 3.0 and 2.7, respectively. However, such an increase could not be ascertained for statistical significance, due to the fewer number of available replicates for the PMA+ samples from the Glut and THPS groups. The fewer number of available replicates is a consequence of less DNA in PMA+ samples, which subsequently resulted in 16S rRNA gene libraries with too few reads for downstream analysis. Overall, our results suggested that none of the biocide or heat treatments substantially affected the Shannon indices of the microbial community when DNA from dead cells was included in the analysis. When PMA was applied to exclude DNA from dead cells, this observation only held true for the no-treatment controls.

**Fig. 5:**
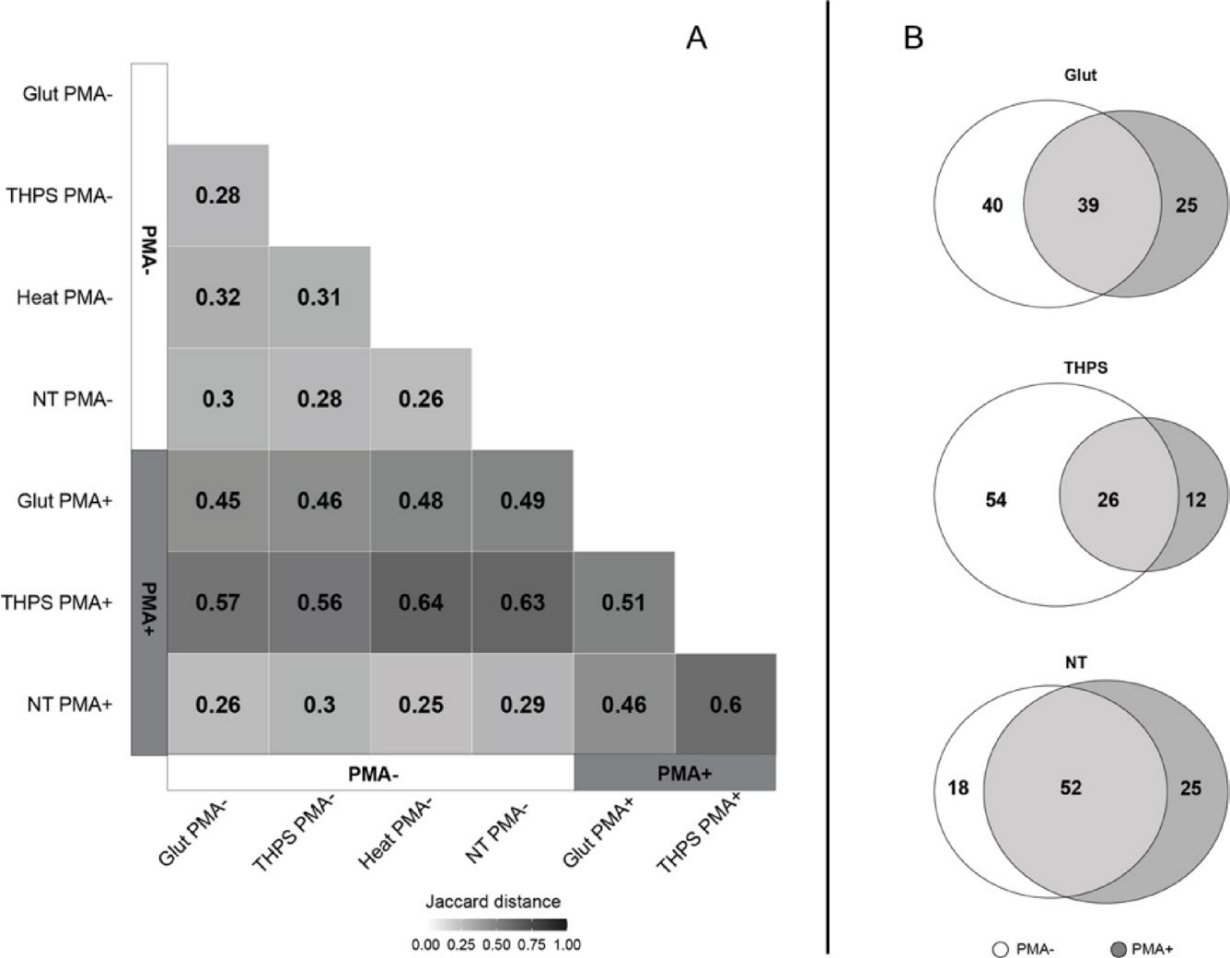
Jaccard distance (**A**) and Venn diagram (**B**) for samples from different groups. This analysis was performed only on samples from which more than 4937 unique sequences were identified, i.e. all PMA- samples and NT PMA+ samples (*N* = 3), 2 of the Glut PMA+ samples, and 1 of the THPS PMA+ samples. In panel A, the distance calculation was based on presence/absence at the genus level. Genera that were present in at least one of the biological replicates from each treatment group of PMA- or PMA+ samples were included in the calculation. In panel B, numbers indicate the number of unique or shared taxa that were present in at least one of the biological replicates from each treatment group of PMA- or PMA+ samples.

We then assessed how PMA affected the *beta diversity* of the microbial communities under different treatments. Here, we calculated two commonly adopted dissimilarity distances, i.e., Jaccard distance and Bray-Curtis distance, to represent the beta diversity between microbial communities. The Jaccard distance quantifies the variance in composition between microbial communities by considering the binary representation of taxa presence or absence within them. Conversely, the Bray-Curtis distance quantifies the compositional dissimilarity across microbial communities based on the relative abundance of taxa. In both cases, a greater value indicates a lower similarity in composition between the microbial communities in comparison^66,67^. Based on the taxa presence and absence, the Jaccard distances among the biocide-, heat- and no-treatment groups consistently measured ca. 0.3 when PMA was not applied **(**Fig. 5A), suggesting that the microbial communities exhibited a relatively high degree of similarity in compositions among them when DNA from dead cells is not removed. When PMA was applied, the microbial community compositions of PMA+ samples from the NT group remained similar to all PMA- samples, with a Jaccard distance of ca. 0.3. In contrast, the Jaccard distance between PMA+ samples from the Glut or THPS groups and all the PMA- samples increased to more than 0.45 (Fig. 5A). This suggests that the microbial community compositions of the Glut and THPS samples, after removing DNA from dead cells, became less similar to samples that included DNA from dead cells, regardless of the biocide treatments. Notably, the Jaccard distance between the PMA+ samples from the Glut group and those from the THPS group was 0.51 (Fig. 5A**)**, exceeding the benchmark value of ca. 0.3. Therefore, after removing the dead cells, the microbial community compositions between the Glut and THPS samples became less similar to each other, suggesting that the different biocides may select different microbial populations. Within the Glut and THPS groups, only 39 out of 104 (37.5%) and 26 out of 92 (28.2%) of the identified genera were shared between the PMA- and PMA+ samples respectively, in contrast to 52 out of 95 (54.7%) shared within the NT group between the PMA- and PMA+ samples (Fig. 5B). Thus, the removal of DNA from dead cells revealed a higher proportion of unique genera identified in the biocide-treated microbial communities compared to the untreated microbial communities.

**Fig. 6:**
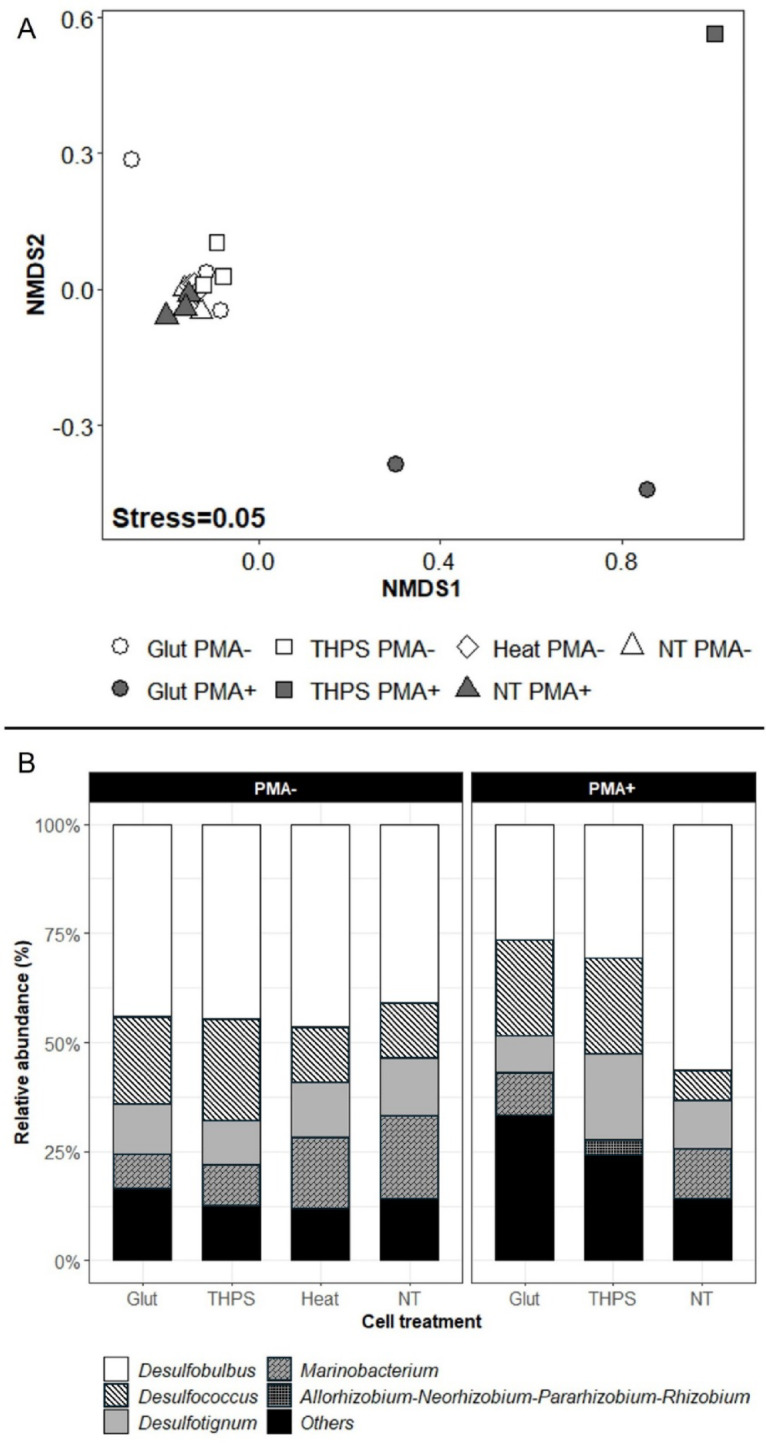
NMDS plot based on Bray-Curtis distance (**A**) and Taxa distribution (**B**) for samples from different groups. Only samples from which more than 4937 unique sequences were detected, i.e. all PMA- samples and NT PMA+ samples (*N* = 3), 2 of the Glut PMA+ samples, and 1 of the THPS PMA+ samples. The calculation was based on the relative abundance at genus level. (A) Points represent individual samples; (B) Stack bars represent the average relative abundance of taxa within the biological replicates from each treatment group of PMA- or PMA+ samples. Only the top four most abundant genera from each group were visualised individually, and all the other genera from each group were aggregated and visualised as “Others”.

Based on the relative abundance of taxa, we calculated the Bray-Curtis distance and represented it through a NMDS plot. This visualisation revealed clear and discernible clustering patterns within the plot. The PMA+ samples from the NT group and all PMA- samples formed a distinct cluster toward the central left of the NMDS plot, while the PMA+ samples from the Glut and THPS groups appeared at the bottom right and top right of the NMDS plot, respectively (Fig. 6A). This observation suggests that the microbial community compositions of the PMA+ samples from the NT group and the PMA- samples across all groups are largely similar, whereas the microbial community compositions of Glut and THPS PMA+ samples differed more from each other and from the rest of samples, as also indicated by the Jaccard distances. Furthermore, the PMA- samples showed a similar taxa distribution at the genus level among the different treatment groups, with *Desulfobulbus* being the most abundant taxon (ca. 45%; Fig. 6B). The PMA+ samples from the Glut and THPS groups showed a lower proportion of *Desulfobulbus* (ca. 25% to 30%) and a higher portion of the less abundant genera (Others, ca. 25%; Fig. 6B).

## Discussion

When evaluating biocide performance, quantifying residual microbial abundance post-treatment is a standard practice, with qPCR assays targeting total DNA (i.e. regardless of cellular viability) being one of the most widely employed molecular tools. These assays rely on the premise that an effective antimicrobial treatment will result in a measurable decline in total microbial DNA, which in turn is used to infer microbial activity or viability following exposure. In our investigation, qPCR analysis performed without the use of PMA revealed a noticeable drop in microbial abundance in samples treated with either Glut or THPS, whilst no such reduction was evident in the heat-killed samples. This result aligns with expectations, as DNA from thermally damaged cells and extracellular nucleic acids often remain intact and are still amplified during PCR, inflating microbial abundance estimates. The apparent reduction in microbial abundance for the Glut and THPS treatments implies that these chemicals may restrict DNA recovery or amplification during downstream analysis. Glutaraldehyde, in particular, is a well-characterized crosslinking agent, known for stabilizing cellular components by forming covalent bonds between proteins via its aldehyde groups^68–70^. It is commonly applied as a fixative in microscopy and preservation methods for cell enumeration^48,49^. At lower doses, it exerts its antimicrobial action by alkylating functional groups such as hydroxyl, amino, and carbonyl moieties, thereby interfering with nucleic acid and protein synthesis^71^. Moreover, it is capable of altering nucleoid structure in bacteria by forming cross-links between DNA and nucleoid-associated proteins, such as HU. Prior studies have reported increased interaction between glutaraldehyde and HU proteins at concentrations up to 500 ppm^72^, potentially explaining the hindered PCR amplification observed in the Glut-treated samples. THPS, another electrophilic compound, exerts toxicity primarily by cleaving disulfide bonds in sulfur-containing amino acids, disrupting protein integrity^10^. While its direct interaction with prokaryotic DNA structures remains unconfirmed, it is reasonable to hypothesize that similar molecular interactions might limit DNA accessibility or integrity in THPS-treated cells, thereby lowering detectable microbial abundance in qPCR. Nonetheless, despite the observed reduction in DNA levels for both Glut and THPS treatments in PMA- samples, these figures did not accurately represent actual microbial activity. Measured microbial cell concentrations remained relatively high, at 5.0 × 10⁴ cells/mL for Glut and 1.3 × 10⁵ cells/mL for THPS (Fig. 2), and sulfate-reducing microorganisms (SRM) accounted for approximately 75% of the detected populations (Fig. 6B). However, no sulfide generation was recorded in these treatments (Fig. 3), suggesting an absence of microbial metabolism. This mismatch between DNA-based cell enumeration (without PMA) and functional activity indicates that biocide performance may be underestimated or misjudged when relying solely on qPCR data without viability discrimination. Such inaccuracies could lead to unnecessary or excessive biocide use in industrial applications.

The use of PMA prior to DNA extraction and subsequent qPCR analysis significantly enhanced the accuracy of microbial abundance measurements by selectively eliminating DNA originating from non-viable cells. This treatment revealed a substantial decline in cell numbers across all intervention groups, including those exposed to glutaraldehyde, THPS, and heat. In these treated samples, the remaining microbial abundance dropped below detectable levels, a contrast to the stable and high DNA levels observed in the untreated controls expectedly dominated by live cells (NT; see Fig. 2). Notably, after applying PMA, the high abundance recorded in untreated samples, approximately 8.1 × 10⁵ cells/mL (NT PMA+; Fig. 2), was consistent with robust biological activity as evidenced by the substantial sulfide accumulation (10 mM over 28 days; Fig. 3). In contrast, samples subjected to Glut, THPS, or heat treatments showed both very low microbial abundance (below 3 × 10³ cells/mL) and negligible sulfide generation (less than 0.1 mM in the same period). This observation is further supported by similar studies that have investigated the influence of disinfectants such as hypochlorite, benzalkonium, monochloramine (MCA), and hydrogen peroxide (H₂O₂) on microbial viability and enumeration. These studies, which focused on both model strains and mixed microbial communities, found that viability indicators such as heterotrophic plate counts (HPC), which estimate the number of viable cells after treatment, generally showed good agreement with microbial abundance determined using PMA-qPCR or PMA-droplet digital PCR (ddPCR) approaches^73,74^. These findings suggest that incorporating PMA enables PCR-based microbial quantification to more accurately reflect the population of viable, metabolically active microorganisms. As such, this approach provides a more meaningful basis for assessing the effectiveness of biocide treatments.

In light of growing concerns about the unintended outcomes of inadequate biocide application, such as fostering microbial populations with increased resistance^18^, there has been a shift in biocide evaluation strategies. The focus is no longer solely on quantifying microbial abundance but has expanded to include broader ecological assessments of microbial communities. This is often achieved through 16S rRNA gene sequencing, which enables the investigation of microbial diversity (alpha and beta) and shifts in community composition during biocide exposure^18,19^. In our investigation, we found that samples from the untreated group (NT), whether treated with PMA or not, displayed consistent microbial diversity and taxonomic profiles (Figs. 4, 5 and 6), suggesting that PMA treatment alone did not introduce detectable changes to community structure. Interestingly, when PMA was not applied, the microbial communities in biocide-treated groups resembled those of the untreated controls in terms of diversity and taxonomic composition (Figs. 4, 5 and 6), expectedly due to the background of dead cells. However, applying PMA revealed marked changes in the compositions in the Glut- and THPS-treated samples, distinguishing them from their PMA- counterparts and the untreated samples. This implies that the biocide interventions had indeed altered key ecological features of the microbial community, but such effects became evident only after the removal of DNA from dead cells via PMA. A key limitation of sequencing-based approaches targeting live cells after effective biocide treatment is the typically low biomass, which can constrain the number of successful replicates and reduce sequencing depth. Our analysis was similarly limited, particularly for the Glut and THPS PMA+ groups, where fewer replicates yielded sufficient reads for interpretation. Future studies may address this issue by increasing the number of replicates, enhancing DNA extraction protocols or optimising sequencing throughput to ensure more robust statistical analysis and a clearer understanding of biocide impacts on surviving microbial populations. Additionally, future studies could explore the application of PMA-based techniques directly in produced water samples without prior cultivation, to better reflect real field conditions and facilitate comparison with conventional industry methods. Finally, it is important to note that the efficacy of PMA depends on sufficient light exposure to activate the dye’s binding to DNA. Therefore, it is more likely to produce false signals in samples containing dark matrices. Although this was not an issue in the current study, it is recommended that dark samples be pre-processed to enhance optical clarity prior to PMA viability staining.

## Conclusions

This study explored the impact of incorporating propidium monoazide (PMA) viability staining into standard DNA-based microbial analysis for assessing biocide performance, particularly applied to engineered systems relevant to the energy sector. Our findings demonstrate that biocide treatments leading to reduced hydrogen sulfide production were also associated with declines in live microbial cell abundance and measurable shifts in microbial diversity and taxonomic composition, but only when PMA was used to exclude DNA from dead cells. Without incorporating PMA, these relationships remained obscured, likely due to interference from residual DNA of dead or damaged organisms. By integrating PMA into existing workflows such as DNA extraction, qPCR, and amplicon sequencing, we achieved a more accurate representation of the live microbial community following biocide exposure. This refinement allowed clearer associations to be drawn between microbial community structure and its potential functional risks, such as sulfide production. The application of PMA introduces minimal additional operational burden in terms of cost, time, or procedural complexity, making it a practical enhancement to current DNA-based monitoring strategies. This approach not only improves the reliability of microbial risk assessments but also strengthens the early detection of ineffective biocide treatments.

## Data Availability

Raw amplicon sequence data (fastq format) generated in this study are available in the NCBI Sequence Read Archive (SRA); BioProject accession number PRJNA1012299.

## References

[1] Wördemann, H. et al. Influence of microbial processes on the operational reliability in a geothermal heat store - results of long-term monitoring at a full scale plant and first studies in a bypass system. *Energy Procedia*. 412–417. 10.1016/j.egypro.2014.10.396 (2014).

[2] Pannekens, M., Kroll, L., Müller, H., Mbow, F. T. & Meckenstock, R. U. Oil reservoirs, an exceptional habitat for microorganisms. *N Biotechnol.*10.1016/j.nbt.2018.11.006 (2019). 30502541 10.1016/j.nbt.2018.11.006 PMC6323355

[3] Morgan, H., Large, D., Bateman, K., Hanstock, D. & Gregory, S. The effect of variable oxygen impurities on microbial activity in conditions resembling geological storage sites. *Energy Procedia*. 3077–3087. 10.1016/j.egypro.2017.03.1437 (2017).

[4] Bixler, G. D. & Bhushan, B. Biofouling: lessons from nature. *Philosophical Trans. Royal Soc. A: Math. Phys. Eng. Sci.*10.1098/rsta.2011.0502 (2012). 10.1098/rsta.2011.0502 22509063

[5] Puentes-Cala, E. et al. Microbiologically influenced corrosion: the gap in the field. *Front. Environ. Sci.*10.3389/fenvs.2022.924842 (2022).

[6] Pinel, I., Biškauskaitė, R., Pal’ová, E., Vrouwenvelder, H. & van Loosdrecht, M. Assessment of the impact of temperature on biofilm composition with a laboratory heat exchanger module. *Microorganisms*. 10.3390/microorganisms9061185 (2021). 10.3390/microorganisms9061185 PMC8229324 34072656

[7] Gieg, L. M., Jack, T. R. & Foght, J. M. Biological souring and mitigation in oil reservoirs. *Appl. Microbiol. Biotechnol.* **92**, 263–282. 10.1007/s00253-011-3542-6 (2011). 21858492 10.1007/s00253-011-3542-6

[8] Johnson, R. J., Folwell, B. D., Wirekoh, A., Frenzel, M. & Skovhus, T. L. Reservoir souring – latest developments for application and mitigation. *J. Biotechnol.* **256**, 57–67. 10.1016/j.jbiotec.2017.04.003 (2017). 28400136 10.1016/j.jbiotec.2017.04.003

[9] McIlwaine, D. B. Oilfield application for biocides. In *Directory of Microbicides for the Protection of Materials: A Handbook* (ed Paulus, W.) 157–175 (Springer Netherlands, 2005).

[10] Kahrilas, G. A., Blotevogel, J., Stewart, P. S. & Borch, T. Biocides in hydraulic fracturing fluids: a critical review of their usage, mobility, degradation, and toxicity. *Environ. Sci. Technol.*10.1021/es503724k (2015). 25427278 10.1021/es503724k

[11] Gardner, L. R. & Stewart, P. S. Action of glutaraldehyde and nitrite against sulfate-reducing bacterial biofilms. *J. Ind. Microbiol. Biotechnol.*10.1038/sj.jim.7000284 (2002). 12483478 10.1038/sj.jim.7000284

[12] Xue, Y. & Voordouw, G. Control of microbial sulfide production with biocides and nitrate in oil reservoir simulating bioreactors. *Front. Microbiol.* **6**, 1387. 10.3389/fmicb.2015.01387 (2015). 26696994 10.3389/fmicb.2015.01387 PMC4672050

[13] Reinsel, M. A., Sears, J. T., Stewart, P. S. & Mclnerney, M. J. Control of microbial souring by nitrate, nitrite or glutaraldehyde injection in a sandstone column. *J. Ind. Microbiol.* **17**, 128–136. 10.1007/BF01570056 (1996).

[14] Hernandez, K., Curtis, T. & Smith, F. A novel approach to using THPS for controlling reservoir souring. In *Paper presented at NACE the CORROSION 2011, Houston, Texas, March 2011*, 1–11. (2011).

[15] Enning, D., Smith, R., Stolle, J. & Hornemann, J. Evaluating the efficacy of weekly THPS and glutaraldehyde batch treatment to control severe microbial corrosion in a simulated seawater injection system. In *Paper Presented at the CORROSION 2016, Vancouver, British Columbia, Canada, March 2016*, 1–15 (2016).

[16] Skovhus, T. L. Biocide testing against microbes. In *Biofouling Methods*, 76–86. 10.1002/9781118336144.ch3 (2014).

[17] Tidwell, T. J., Keasler, V. S. & de Paula, R. How production chemicals can influence microbial susceptibility to biocides and impact mitigation strategies. In *Microbiologically Influenced Corrosion in the Upstream Oil and Gas Industry*, 379–392 (2017).

[18] Shi, X., Oliveira, D. A. F., Holsten, L., Steinhauer, K. & de Rezende, J. R. Long-term biocide efficacy and its effect on a souring microbial community. *Appl. Environ. Microbiol.*10.1128/aem.00842-21 (2021). 34160245 10.1128/AEM.00842-21 PMC8357289

[19] Shi, X., de 637 Rezende, J. R. & Sorbie, K. Microbial ecology metrics to assess the effect of biocide on souring control and improve souring modelling. In *Paper Presented at the SPE International Oilfield Corrosion Conference and Exhibition, Virtual, June 2021*. 10.2118/205037-MS (2021).

[20] Jenneman, G. E. & de Leόn, K. B. Environmental stressors alter the susceptibility of microorganisms to biocides in upstream oil and gas systems. *Int. Biodeterior. Biodegradation*. 10.1016/j.ibiod.2022.105385 (2022).

[21] Emerson, J. B. et al. Schrödinger’s microbes: tools for distinguishing the living from the dead in microbial ecosystems. *Microbiome*10.1186/s40168-017-0285-3 (2017). 28810907 10.1186/s40168-017-0285-3 PMC5558654

[22] Stanley, P. E. A review of bioluminescent ATP techniques in rapid microbiology. *J. Biolumin. Chemilumin.*10.1002/bio.1170040151 (1989). 2678922 10.1002/bio.1170040151

[23] Selan, L., Berlutti, F., Passariello, C., Thaller, M. C. & Renzini’, G. Reliability of a bioluminescence ATP assay for detection of bacteria. *J. Clin. Microbiol.*10.1128/jcm.30.7.1739-1742.1992 (1992). 10.1128/jcm.30.7.1739-1742.1992 PMC265373 1629329

[24] Johnson, D. R., Lee, P. K. H., Holmes, V. F. & Alvarez-Cohen, L. An internal reference technique for accurately quantifying specific mRNAs by real-time PCR with application to the TceA reductive dehalogenase gene. *Appl. Environ. Microbiol.*10.1128/aem.71.7.3866-3871.2005 (2005). 16000799 10.1128/AEM.71.7.3866-3871.2005 PMC1169012

[25] Nocker, A., Cheung, C. Y. & Camper, A. K. Comparison of propidium monoazide with ethidium monoazide for differentiation of live vs. dead bacteria by selective removal of DNA from dead cells. *J. Microbiol. Methods*. 10.1016/j.mimet.2006.04.015 (2006). 16753236 10.1016/j.mimet.2006.04.015

[26] Nocker, A., Sossa-Fernandez, P., Burr, M. D. & Camper, A. K. Use of propidium monoazide for live/dead distinction in microbial ecology. *Appl. Environ. Microbiol.*10.1128/aem.02987-06 (2007). 17586667 10.1128/AEM.02987-06 PMC1951001

[27] Fraisse, A. et al. Discrimination of infectious and heat-treated norovirus by combining platinum compounds and real-time RT-PCR. *Int. J. Food Microbiol.*10.1016/j.ijfoodmicro.2018.01.015 (2018). 29421360 10.1016/j.ijfoodmicro.2018.01.015

[28] Randazzo, W., Piqueras, J., Rodríguez-Díaz, J., Aznar, R. & Sánchez, G. Improving efficiency of viability-qPCR for selective detection of infectious HAV in food and water samples. *J. Appl. Microbiol.*10.1111/jam.13519 (2018). 28649706 10.1111/jam.13519

[29] Rey, M. et al. Evaluation of PMA-qPCR methodology to detect and quantify viable Shiga toxin-producing Escherichia coli in beef burgers. *J. Food Process. Preserv*. 10.1111/jfpp.15338 (2021).

[30] Elizaquível, P., Aznar, R. & Sánchez, G. Recent developments in the use of viability dyes and quantitative PCR in the food microbiology field. *J. Appl. Microbiol.*10.1111/jam.12365 (2014). 24119073 10.1111/jam.12365

[31] Rousseau, A. et al. Evaluation of propidium monoazide–based qPCR to detect viable oocysts of Toxoplasma gondii. *Parasitol. Res.*10.1007/s00436-019-06220-1 (2019). 30729299 10.1007/s00436-019-06220-1

[32] Barbau-Piednoir, E. et al. Evaluation of viability-qPCR detection system on viable and dead Salmonella serovar enteritidis. *J. Microbiol. Methods*. 10.1016/j.mimet.2014.06.003 (2014). 24927988 10.1016/j.mimet.2014.06.003

[33] Randazzo, W., López-Gálvez, F., Allende, A., Aznar, R. & Sánchez, G. Evaluation of viability PCR performance for assessing norovirus infectivity in fresh-cut vegetables and irrigation water. *Int. J. Food Microbiol.*10.1016/j.ijfoodmicro.2016.04.010 (2016). 27085970 10.1016/j.ijfoodmicro.2016.04.010

[34] Li, D. et al. Quantification of viable bacteria in wastewater treatment plants by using propidium monoazide combined with quantitative PCR (PMA-qPCR). *J. Environ. Sci. (China)*. 10.1016/s1001-0742(13)60425-8 (2014). 25076521 10.1016/s1001-0742(13)60425-8

[35] Lee, A. S. et al. A novel propidium monoazide-based PCR assay can measure viable uropathogenic E. coli in vitro and in vivo. *Front. Cell. Infect. Microbiol.*10.3389/fcimb.2022.794323 (2022). 35178354 10.3389/fcimb.2022.794323 PMC8844370

[36] Latka, A. et al. Optimization of propidium monoazide qPCR (viability-qPCR) to quantify the killing by the Gardnerella-specific endolysin PM-477, directly in vaginal samples from women with bacterial vaginosis. *Antibiotics*10.3390/antibiotics11010111 (2022). 35052988 10.3390/antibiotics11010111 PMC8773202

[37] Han, S. et al. Detection of Clavibacter michiganensis subsp. michiganensis in viable but nonculturable state from tomato seed using improved qPCR. *PLoS One*. 10.1371/journal.pone.0196525 (2018). 29723290 10.1371/journal.pone.0196525 PMC5933903

[38] Carini, P. et al. Relic DNA is abundant in soil and obscures estimates of soil microbial diversity. *Nat. Microbiol.*10.1038/nmicrobiol.2016.242 (2016). 27991881 10.1038/nmicrobiol.2016.242

[39] Vaishampayan, P. et al. New perspectives on viable microbial communities in low-biomass cleanroom environments. *ISME J.*10.1038/ismej.2012.114 (2013). 23051695 10.1038/ismej.2012.114 PMC3554398

[40] Papanicolas, L. E. et al. Bacterial viability in faecal transplants: which bacteria survive? *EBioMedicine*. 10.1016/j.ebiom.2019.02.023 (2019). 10.1016/j.ebiom.2019.02.023 PMC6444077 30796005

[41] Rogers, G. B. et al. Reducing bias in bacterial community analysis of lower respiratory infections. *ISME J.*10.1038/ismej.2012.145 (2013). 10.1038/ismej.2012.145 PMC3603400 23190732

[42] García-Fontana, C. et al. A new physiological role for the DNA molecule as a protector against drying stress in desiccation-tolerant microorganisms. *Front. Microbiol.*10.3389/fmicb.2016.02066 (2016). 10.3389/fmicb.2016.02066 PMC5177630 28066383

[43] Ramírez, G. A., Jørgensen, S. L., Zhao, R. & D’Hondt, S. Minimal influence of extracellular DNA on molecular surveys of marine sedimentary communities. *Front. Microbiol.*10.3389/fmicb.2018.02969 (2018). 30564217 10.3389/fmicb.2018.02969 PMC6288230

[44] Hirohara, T., Tsuri, K., Miyagawa, K., Paine, R. T. R. & Yamanaka, H. The application of PMA (propidium monoazide) to different target sequence lengths of zebrafish eDNA: a new approach aimed toward improving environmental DNA ecology and biological surveillance. *Front. Ecol. Evol.*10.3389/fevo.2021.632973 (2021).

[45] Bijlsma, R. & Loeschcke, V. Environmental stress, adaptation and evolution: an overview. *J. Evol. Biol.*10.1111/j.1420-9101.2005.00962.x (2005). 16033544 10.1111/j.1420-9101.2005.00962.x

[46] Vikram, A., Bomberger, J. M. & Bibby, K. J. Efflux as a glutaraldehyde resistance mechanism in Pseudomonas fluorescens and Pseudomonas aeruginosa biofilms. *Antimicrob. Agents Chemother.*10.1128/aac.05152-14 (2015). 25824217 10.1128/AAC.05152-14 PMC4432172

[47] Maillard, J-Y. Mechanisms of bacterial resistance to microbicides. In *Principles and Practice of Disinfection, Preservation and Sterilization. Russell, Hugo & Ayliffe’s*, 108–120 10.1002/9781118425831.ch6a (2013).

[48] Kamiya, E., Izumiyama, S., Nishimura, M., Mitchell, J. G. & Kogure, K. Effects of fixation and storage on flow cytometric analysis of marine bacteria. *J. Oceanogr.*10.1007/s10872-007-0008-7 (2007).

[49] Vignola, M., Werner, D., Hammes, F., King, L. C. & Davenport, R. J. Flow cytometric quantification of microbial cells on sand from water biofilters. *Water Res.*10.1016/j.watres.2018.05.053 (2018). 29940363 10.1016/j.watres.2018.05.053

[50] Widdel, F. & Bak, F. Gram negative mesophilic sulfate-reducing bacteria. In *The Prokaryotes*. 3352–3378. (1992).

[51] Cord-Ruwisch, R. A quick method for the determination of dissolved and precipitated sulfides in cultures of sulfate-reducing bacteria. *J. Microbiol. Methods*. 10.1016/0167-7012(85)90005-3 (1985).

[52] Griffiths, R. I., Whiteley, A. S., O’donnell, A. G. & Bailey, M. J. Rapid method for coextraction of DNA and RNA from natural environments for analysis of ribosomal DNA- and rRNA-based microbial community composition. *Appl. Environ. Microbiol.*10.1128/aem.66.12.5488-5491.2000 (2000). 10.1128/aem.66.12.5488-5491.2000 PMC92488 11097934

[53] Lane, D. J. 16S 764 /23S rRNA sequencing. In *Nucleic Acid Techniques in Bacterial Systematics*, 205–248 (1991).

[54] Ruijter, J. M. et al. Amplification efficiency: linking baseline and bias in the analysis of quantitative PCR data. *Nucleic Acids Res.*10.1093/nar/gkp045 (2009). 10.1093/nar/gkp045 PMC2665230 19237396

[55] Daghio, M. et al. Anodes stimulate anaerobic toluene degradation via sulfur cycling in marine sediments. *Appl. Environ. Microbiol.* **82**, 297–307 (2016). 26497463 10.1128/AEM.02250-15 PMC4702649

[56] Ramakers, C., Ruijter, J. M., Lekanne Deprez, R. H. & Moorman, A. F. M. Assumption-free analysis of quantitative real-time polymerase chain reaction (PCR) data. *Neurosci. Lett.*10.1016/s0304-3940(02)01423-4 (2003). 12618301 10.1016/s0304-3940(02)01423-4

[57] Brankatschk, R., Bodenhausen, N., Zeyer, J. & Burgmann, H. Simple absolute quantification method correcting for quantitative PCR efficiency variations for microbial community samples. *Appl. Environ. Microbiol.*10.1128/aem.07878-11 (2012). 22492459 10.1128/AEM.07878-11 PMC3370567

[58] Stoddard, S. F., Smith, B. J., Hein, R., Roller, B. R. K. & Schmidt, T. M. RrnDB: improved tools for interpreting rRNA gene abundance in bacteria and archaea and a new foundation for future development. *Nucleic Acids Res.*10.1093/nar/gku1201 (2015). 25414355 10.1093/nar/gku1201 PMC4383981

[59] Kozich, J. J., Westcott, S. L., Baxter, N. T., Highlander, S. K. & Schloss, P. D. Development of a dual-index sequencing strategy and curation pipeline for analyzing amplicon sequence data on the miseq illumina sequencing platform. *Appl. Environ. Microbiol.*10.1128/AEM.01043-13 (2013). 10.1128/AEM.01043-13 PMC3753973 23793624

[60] Caporaso, J. G. et al. Global patterns of 16S rRNA diversity at a depth of millions of sequences per sample. *Proc. Natl. Acad. Sci.*10.1073/pnas.1000080107 (2011). 20534432 10.1073/pnas.1000080107 PMC3063599

[61] Klindworth, A. et al. Evaluation of general 16S ribosomal RNA gene PCR primers for classical and next-generation sequencing-based diversity studies. *Nucleic Acids Res.*10.1093/nar/gks808 (2013). 10.1093/nar/gks808 PMC3592464 22933715

[62] Schloss, P. D. et al. Introducing mothur: open source, platform-independent, community-supported software for describing and comparing microbial communities. *Appl. Environ. Microbiol.*10.1128/aem.01541-09 (2009). 19801464 10.1128/AEM.01541-09 PMC2786419

[63] McMurdie, P. J. & Holmes, S. Phyloseq: an R package for reproducible interactive analysis and graphics of microbiome census data. *PLoS One*. 10.1371/journal.pone.0061217 (2013). 23630581 10.1371/journal.pone.0061217 PMC3632530

[64] Oksanen, J. et al. vegan: Community Ecology Package. R package version 2.6 (2022).

[65] Shannon, C. E. A mathematical theory of communication. *Bell Syst. Tech. J.*10.1002/j.1538-7305.1948.tb01338.x (1948).

[66] Jaccard, P. The distribution of the flora in the alpine zone. *New Phytol.*10.1111/j.1469-8137.1912.tb05611.x (1912).

[67] Bray, J. R. & Curtis, J. T. An ordination of the upland forest communities of Southern Wisconsin. *Ecol. Monogr.*10.2307/1942268 (1957).

[68] Hu, W., Murata, K. & Zhang, D. Applicability of LIVE/DEAD BacLight stain with glutaraldehyde fixation for the measurement of bacterial abundance and viability in rainwater. *J. Environ. Sci. (China)*. 10.1016/j.jes.2016.05.030 (2017). 28115131 10.1016/j.jes.2016.05.030

[69] Ross, P. W. A new disinfectant. *J. Clin. Pathol.*10.1136/jcp.19.4.318 (1966). 10.1136/jcp.19.4.318 PMC473273 5929332

[70] Gorman Eileen, M., Scott, S. P. & Russell, A. D. Antimicrobial activity. Uses and mechanism of action of glutaraldehyde. *J. Appl. Bacteriol.*10.1111/j.1365-2672.1980.tb01217.x (1980). 10.1111/j.1365-2672.1980.tb01217.x 6780502

[71] McGucken, P. V. & Woodside, W. Studies on the mode of action of glutaraldehyde on Escherichia coli. *J. Appl. Bacteriol.*10.1111/j.1365-2672.1973.tb04123.x (1973). 4201827 10.1111/j.1365-2672.1973.tb04123.x

[72] Bhowmick, T. et al. Targeting Mycobacterium tuberculosis nucleoid-associated protein HU with structure-based inhibitors. *Nat. Commun.*10.1038/ncomms5124 (2014). 24916461 10.1038/ncomms5124

[73] Yang, Y. et al. Effects of monochloramine and hydrogen peroxide on the bacterial community shifts in biologically treated wastewater. *Chemosphere*. 10.1016/j.chemosphere.2017.09.087 (2017). 10.1016/j.chemosphere.2017.09.087 28950119

[74] Nocker, A., Sossa, K. E. & Camper, A. K. Molecular monitoring of disinfection efficacy using propidium monoazide in combination with quantitative PCR. *J. Microbiol. Methods*. 10.1016/j.mimet.2007.04.014 (2007). 17544161 10.1016/j.mimet.2007.04.014

